# Structural Equation Model (SEM) of Stroke Mortality in Spanish Inpatient Hospital Settings: The Role of Individual and Contextual Factors

**DOI:** 10.3389/fneur.2019.00498

**Published:** 2019-05-17

**Authors:** Jesús de la Fuente, Juan Manuel García-Torrecillas, Giulliana Solinas, María Mar Iglesias-Espinosa, Angélica Garzón-Umerenkova, Javier Fiz-Pérez

**Affiliations:** ^1^Educational Psychology, School of Education and Psychology, University of Navarra, Pamplona, Spain; ^2^Educational Psychology, School of Psychology, University of Almería, Almería, Spain; ^3^Emergency and Research Unit, University Hospital Torrecárdenas, Almería, Spain; ^4^CIBER of Epidemiology and Public Health (CIBERESP), Madrid, Spain; ^5^Biotechnology, Department of Medicine, University of Sassari, Sassari, Italy; ^6^Department of Neurology and Neurophysiology, University Hospital Torrecárdenas, Almería, Spain; ^7^Fundación Universitaria Konrad Lorenz, Bogotá, Colombia; ^8^Organizational and Developmental Psychology, Università Europea di Roma, Rome, Italy

**Keywords:** stroke, mortality, structural equation model, predictive model, inpatient hospital

## Abstract

**Introduction:** Traditionally, predictive models of in-hospital mortality in ischemic stroke have focused on individual patient variables, to the neglect of in-hospital contextual variables. In addition, frequently used scores are betters predictors of risk of sequelae than mortality, and, to date, the use of structural equations in elaborating such measures has only been anecdotal.

**Aims:** The aim of this paper was to analyze the joint predictive weight of the following: (1) individual factors (age, gender, obesity, and epilepsy) on the mediating factors (arrhythmias, dyslipidemia, hypertension), and ultimately death (exitus); (2) contextual in-hospital factors (year and existence of a stroke unit) on the mediating factors (number of diagnoses, procedures and length of stay, and re-admission), as determinants of death; and (3) certain factors in predicting others.

**Material and Methods:** Retrospective cohort study through observational analysis of all hospital stays of Diagnosis Related Group (DRG) 14, non-lysed ischemic stroke, during the time period 2008–2012. The sample consisted of a total of 186,245 hospital stays, taken from the Minimum Basic Data Set (MBDS) upon discharge from Spanish hospitals. MANOVAs were carried out to establish the linear effect of certain variables on others. These formed the basis for building the Structural Equation Model (SEM), with the corresponding parameters and restrictive indicators.

**Results:** A consistent model of causal predictive relationships between the postulated variables was obtained. One of the most interesting effects was the predictive value of contextual variables on individual variables, especially the indirect effect of the existence of stroke units on reducing number of procedures, readmission and in-hospital mortality.

**Conclusion:** Contextual variables, and specifically the availability of stroke units, made a positive impact on individual variables that affect prognosis and mortality in ischemic stroke. Moreover, it is feasible to determine this impact through the use of structural equation methodology. We analyze the methodological and clinical implications of this type of study for hospital policies.

## Introduction

### Prevalence of Ischemic Stroke

According to the WHO, ischemic stroke (IS) is the third leading cause of death in Western countries, and the first cause of disability in adults, in addition to having a high morbimortality load (1). In the USA alone, there are 800,000 persons every year who experience a stroke incident, either first-time or recurrent. The age-adjusted mortality rate in the most recent American studies has shown that stroke is a direct, underlying cause in 36.2 of every 100,000 exitus per year (2).

In *Europe*, as of today, the age-standardized incidence of stroke falls between 95 and 290 episodes per 100,000 inhabitants, with 1-month mortality between 10 and 35%; stroke represents the second leading cause of morbidity and disability (3). The present situation in Europe is rising incidence among young adults, despite the decreasing trend worldwide. Mortality is not the only parameter of interest; 33% will require readmission to hospital, 7–13% will have another episode, moderate cognitive decline will affect 35–47% and dementia, 7–23% (3). Consequently, morbidity load as well as mortality are pressor elements in this population; they have important repercussions today, and in the case of Europe, can only be expected to worsen in coming years.

In *Spain*, mortality due to cardiovascular causes and stroke in particular began to decline in 1973, thanks to improved attention to cardiovascular risk factors associated with greater stroke mortality, as well as to diagnostic and therapeutic advances in the earliest phases of care. Very heterogeneous values of incidence in Spain have been reported, as seen in the study by Lópoez-Pousa et al. (4). Subsequently, the Iberictus study, led by the Spanish Society of Neurology, allowed access to more up-to-date, quality data, showing an incidence of 118 cases per 100,000 inhabitants per year. In-hospital mortality was also reported as 4% (5, 6). Nonetheless, rising mortality rates are to be expected in the future, due to pronounced aging of the population and the increased prevalence of risk factors in an increasingly elderly population (5). Currently, ischemic stroke is the second leading cause of death in Spain in the general population and the first cause of death in women (6); according to clinical records in our country, it represents 12.9% of total deaths (7).

### Risk Factors for Developing a Stroke

The risk factors associated with stroke incidence and mortality are well-known. These factors can be divided into *personal factors* (related to the patient, regardless of modifiability) and *contextual factors*, which are usually associated with availability of specific resources, shorter time to care, and the establishment of specific plans for stroke care (8, 9).

The most notable, prevalent *individual risk factors* for developing a stroke include hypertension (HTN), Diabetes Mellitus (DM), abnormal heart rhythm (especially atrial fibrillation), hyperlipidemia and hypertriglyceridemia, liver disease, smoking, sedentary lifestyle and finally nutritional and genetic factors (2, 10). Sleep apnea and certain psychosocial factors have also been associated. The factors mentioned not only increase incidence, but also subsequent mortality (11). Predictors of poor evolution include the severity of the initial stroke, measured on the National Institute of Health Stroke Scale (NIHSS) or Canadian Neurological Scale (CNS); existence of diabetes mellitus; large or pronounced drops in blood pressure; body temperature; certain coagulation markers; and inflammation and glycemia at hospital admission (12).

In addition to individual factors, there are other important prognosis factors that have seldom been studied in conjunction with the individual factors; we will call these *contextual risk factors*. The existence of a comprehensive plan of action—which maximizes and optimizes patient care from the time of hospital arrival—has been shown to have beneficial results for patients who have suffered an acute stroke, increasing their probability of recovery (13). Over the past 20 years, not only the change in preventive action, but also early, regulated response that follows the most advanced quality standards, and the creation of specific stroke care units, have been shown to bring about a significant decrease in stroke mortality and sequelae.

### The Construction of Probabilistic Prediction Models

Extensive work has been done in detecting the risk factors of developing an ischemic event and in estimating the likelihood of death or of sequelae (7). Specifically, work by Smith et al. (14) produced predictive models of in-hospital mortality, whether for ischemic or hemorrhagic stroke, using a limited number of variables; excellent estimated discriminative capacity was attained. Other highly interesting work has shown a successful methodology for elaborating predictive models of stroke (15).

Since the creation of stroke units, there have been numerous studies where these units demonstrate a decrease in mortality and disability, in comparison to the administration of conventional care (Cochrane Database of Systematic Reviews, 2013). More recently, their cost-effectiveness and a shortened average length of stay have also been demonstrated (16).

### Aims and Hypotheses

A large part of the literature has focused on individual prognosis factors, while other authors have assessed isolated contextual elements, especially the availability of stroke units. To date, there is insufficient evidence that combines both types of variables and explores their interrelations using a structural, hierarchical equation methodology.

Consequently, our main *objective* was to establish interdependent and predictive relationships among the variables that are most often identified in association with pathogenesis and development of stroke, and the main dependent variables (mortality and readmission to hospital). Specifically, and original to this study, we evaluated the role of certain process and context variables, and how they acted as intermediate, modulating variables in the non-linear relationship between predictive variables and outcome variables.

In order to address the main objective, the initial *hypothesis* states that each individual variable defined in the linear model (primarily age, gender, obesity, and epilepsy) and each contextual variable (year, existence of stroke units) would have a statistically significant effect on the intermediate variables of the previously established linear model, whether individual variables (arrhythmias, dyslipidemia, and hypertension) or contextual (length of stay, number of diagnoses, and procedures). These in turn would have a significant effect on the two final, dependent variables, namely, readmissions, and mortality.

## Materials and Methods

### Participants

#### Type of Study

A retrospective cohort study using analytical observation of all hospital stays of the Diagnosis Related Group (GRD) 14—non-lysed ischemic stroke—during the time period 2008–2012. All hospital stays of patients age 24 or older were included.

#### Scope

The study was carried out within the Spanish National Healthcare System (NHS, Spain), a decentralized structure across 17 autonomous regions with their respective regional healthcare systems. Each of the Autonomous Systems has its own structure, with Basic Healthcare Zones grouped in turn into Primary Care Districts and Hospitals. This system is the same throughout the country, despite the drawback of frequent failures in inter-region communication. Healthcare within this network is free of charge; costs are borne by the different regional governments.

#### Information Source, Sample, and Case Selection

The source of information was the Spanish Minimum Basic Hospital Discharge Dataset, made available by the Ministry of Health, Consumerism and Social Policies. A total of 186,245 hospital stays were analyzed. Diagnostic and procedural coding followed the *International Classification of Diseases, Ninth Revision, Clinical Modification* (ICD9MC). The selection criteria consisted of identifying the patient stays that were discharged under DRG 14 (AP-DRG classifier, version 21). This diagnostic group includes exclusively those patients admitted for ischemic stroke who undergo medical treatment, but not fibrinolysis or mechanical reperfusion; consequently, this DRG defines a very specific, select group of patients. As in the relevant bibliography, the total group of hospital stays was then limited to patients over the age of 24, given the small incidence and prevalence of these events in younger persons. Additionally, outlier hospital stays were filtered out according to the classical method that defines outliers with the formula T2 = Q3+1.5(Q3-Q1), where Q identifies the third and first quartiles and T2 is the maximum value of the stay that results from applying the formula. Using this methodology, patients with stays longer than 21 days were identified and excluded.

## Procedure

This project has been approved by the Clinical Ethics Committee of the Province of Almeria, Complejo Hospitalario Torrecardenas, Andalusian Health Service, Ministry of Health, Andalusia (Spain).

## Data Analysis

### Variables and Analysis Schema

The schema of analysis identified two axes for studying relations and associations between variables. On one hand, variables were classified into two large dimensions in each episode: individual and context dimensions. The *context* variables were identified as year, existence of a stroke unit, length of stay, total count of diagnoses and procedures at discharge, and any readmissions; the remaining variables were considered individual variables (Table 1). On the other hand, our second axis of analysis classified variables as independent variables, intermediate/process variables, or outcome/dependent variables—regardless of the dimension to which they belonged.

**Table 1:** Classification of the constituent variables of the model along the two axes of coding and analysis.

	**Previous or independent var**.	**Intermediate var**.	**Final, dependent or outcome var**.
**VARIABLES CLASSIFIED ACCORDING TO TWO AXES OF ANALYSIS**			
Individual variables	Age	Arrhythmias	Mortality
	Gender	Dyslipidemia	
	Obesity	HTN	
	Epilepsy		
Contextual variables	Year	Stay	Readmission
	Stroke unit	N° of Diagnoses	
		N° Procedures	
**CODING AND CLASSIFICATION OF INDIVIDUAL VS. CONTEXT VARIABLES**			
**Individual variables**	Renal insufficiency (%)		
Age (years)	Anemia (%)		
Gender (M/F) (%)	Pulmonary embolism (%)		
Obesity (%)	Heart Failure (%)		
Epilepsy (%)	Acute Respiratory Insufficiency (%)		
Arrythmias (%)	Topographic location of stroke		
Dyslipidemia (%)	Exitus (%)		
Hypertension (%)	**In-hospital contextual variables**		
Diabetes (%)	Year (2008 to 2012)		
COPD (%)	Stroke Unit Available (%)		
Ischemic Cardiopathy (%)	Length of stay (days)		
Valvulopathy (%)	NDX (quantitative)		
Myocardiopathy (%)	NPR (quantitative)		
Congenital Cardiopathy (%)	Readmission at 30 days (%)		

*NDX, Number of diagnoses at discharge; NPR, Number of procedures at discharge; COPD, Chronic obstructive pulmonary disease*.

The main dependent variable in the individual dimension was in-hospital mortality. Secondarily, readmissions were also analyzed as a dependent variable in the context dimension. According to the second axis of analysis, both individual and contextual variables were classified as outcomes (exitus and readmission), intermediate or process variables (arrhythmias, dyslipidemia, HTN, length of stay, NDX, and NPR) or initial variables (age, gender, obesity, epilepsy, year, stroke unit) (Table 1). One must keep in mind that the variables that make up the secondary diagnoses cannot always be identified differentially as complications that occurred during hospitalization or as pre-existing patient comorbidities, such as epilepsy.

In order to make the Year variable (6 categories) more homogeneous, the derived variable “Year Gp” was obtained by establishing three bienniums.

Sociodemographic information was obtained from the variables year, age, gender, and Autonomous Region. Administrative elements were assessed through the variables length of stay, readmission within 30 days for the same DRG, type of admission (emergency vs. scheduled), and type of discharge (alive vs. exitus). We used the number of diagnoses at discharge (NDX) as a proxy variable for the patient's comorbidity, and the number of procedures at discharge (NPR) to estimate the procedural complexity of each episode and the main clinical comorbidities associated with ischemic processes (Table 2).

**Table 2:** Principal and partial effects of independent variables (presage) on dependent variables (process).

**Main effect**		**F (Pillai)**	**df**	***p < ***	***np^**2**^***	**Power**
AGE		7.748	42.000	0.000	0.000	1.00^*^
OBESITY		13.291	7.000	0.000	0.001	1.00^*^
EPILEPSY		7.785	7.000	0.000	0.000	1.00^*^
YEAR		2.009	28.000	0.001	0.000	0.999^*^
UNIT		9.428*b*	7.000	0.000	0.000	1.00^*^
**IV**	**DV**		**F (Pillai)**	***p < ***	***np**^**2**^*	**Power**
**PARTIAL EFFECT (ONLY SIGNIFICANT PARTIAL EFFECTS)**						
GENDER ^*^						
OBESITY ^*^						
UNIT	ARRHYTHMIAS		3.811	0.051	0.000	0.497^*^

General Linear model.

* *Observed power of effect (only statistically significant). Full table provided in the Complimentary / Supplementary Material*.

For each of the hospitalization episodes, the total number of diagnoses was calculated (including both new comorbidities and pre-existing comorbidities at the time of admission) and coded into 14 fields of variables assigned for that purpose. In this way, diagnosis number 1 is the one that motivates the admission and the rest of the diagnoses are recorded sequentially, some as derivatives of others, until completing the entire spectrum of pathology that existed in each event.

### Statistical Analysis

For the statistical analysis, variables were treated as follows, according to the dimension being analyzed: (1) first, the initial variables were the independent variables (IV), and the process and outcome variables were dependent (DV); (2) second, the independent process variables were the IV and the outcome variables *exitus* (death) and readmission were the DV.

Two types of analysis were carried out in order to determine which variables to include in the structural linear model. First, *bivariate analysis* was carried out; Student's *t*-test was used to test the equality of means hypothesis for independent samples or analysis of variance. In cases where they could not be applied, the Mann-Whitney or Kruskall-Wallis non-parametric *U* was applied, as appropriate. The Chi-square test was used for comparison of qualitative variables. Relationships between quantitative variables were determined through Pearson or Spearman correlations. Second, *uni- and multi-variate inferential analysis* was carried out between the variables established in the rational model. Inferential statistical analyses (multivariate analysis, MANOVAs) were carried out using SPSS (v. 23.0) for Windows.

Once the variables were identified, the empirical model of structural equations was finally developed. AMOS (v. 23.0) for Windows was used to construct the structural prediction model—specifically, to verify the structural linear prediction hypothesis (path analysis). To interpret the confirmatory factor analysis (CFA) and the structural equation model (SEM) fit, we focused on the Comparative Fit Index (CFI) and the Root Mean Square Error of Approximation (RMSEA). CFI values equal to or greater than 0.90 and 0.95, respectively, were taken to indicate acceptable and close fit to the data (17). RMSEA values equal to or below 0.05 and 0.08 were taken to indicate close and acceptable levels of fit, respectively (18). Keith (19) proposed the following beta coefficients as research benchmarks for direct effects: less than 0.05 is considered too small to be meaningful, above 0.05 is small but meaningful, above 0.10 is moderate, and above 0.25 is large. For indirect effects, we used Kenny's (20) definition of an indirect effect as the product of two effects; using Keith's benchmarks above, we proposed a small indirect effect = 0.003, moderate = 0.01, and large = 0.06, values that are significant in the sphere of education.

## Results

### Basic Descriptive Results

The sample was composed of 186,245 hospital stays between the years 2008 and 2012. There were a total of 12,800 exitus during hospitalization. Over the study period, the death rate declined from 7.3% in 2008 to 6.5% in 2012, for an average rate of 6.9% for the whole period. Mean age of the sample was 79.92 (SD 12.54) years, with a mean hospital stay of 7.54 (SD 4.54) days, and 3.27 (SD 2.45) was the mean number of procedures applied. The mean number of diagnoses at discharge was 6.91 (SD 2.95), and 4.8% of the sample were in a readmission situation under the same DRG. Table 3 shows the distribution of the main variables by year.

**Table 3:** Baseline description over the period.

	**2008**	**2009**	**2010**	**2011**	**2012**
Age (SD) years	73.73 (12.31)	73.89 (12.44)	73.99 (12.54)	73.88 (12.72)	74.09 (12.69)
Hospital stay (SD) days	7.89 (4.67)	7.74 (4.59)	7.58 (4.53)	7.32 (4.45)	7.17 (4.42)
NDX (SD) diagnoses	6.26 (2.73)	6.55 (2.83)	6.82 (2.91)	7.32 (3.00)	7.60 (3.09)
NPR (SD) procedures	3.05 (2.24)	3.16 (2.43)	3.31 (2.51)	3.42 (2.55)	3.42 (2.50)
Female gender (%)	46.4	47	46.9	46.5	46.7
Readmission (%)	4.9	3.8	4.8	4.7	4.8
Emergency admission (%)	97.4	97.2	97.1	97.2	96.6

*NDX, Number of diagnoses at discharge; NPR, Number of procedures at discharge*.

### Inferential Relations Among Variables

First, we assessed the effects of the individual-related IVs (age, gender, obesity, and epilepsy) and context-related IVs (year and existence of a stroke unit) on the intermediate individual variables (arrhythmias, dyslipidemia, and HTN) and intermediate contextual variables (length of stay, NDX, and NPR).

Findings showed a significant effect from each of the IVs (both contextual and individual) on the intermediate variables mentioned, except in the case of gender. No statistically significant, main interaction appeared. There were also numerous significant partial effects of each independent variable on the dependent variables; these are marked with an asterisk to the right in Table 2.

Afterward, we analyzed the effect of the individual IVs and the contextual IVs on the main dependent, individual variable (exitus). Uni- and multi-variate analyses showed a significant main effect of all the individual and contextual factors mentioned, except gender; in addition, evidence showed that the discrete factors with the greatest effect on mortality were age and epilepsy, followed by the existence of stroke units. Moreover, certain variables produced several significant interaction effects on mortality (Table 4), with the greatest observed power detected for the interactions of (Year^*^Stroke unit), (Age^*^Epilepsy^*^Year), and finally the interaction of *(Year* ^*^Gender^*^Obesity^*^StrokeUnit), the latter demonstrating great explanatory power. The most relevant variable, common to two of the interactions detected, was the existence of stroke units. Likewise, the intermediate or process variables, whether related to the individual (arrhythmias, dyslipidemia, and HTN) or to the context (length of stay, NDX, and NPR), had a clear effect on mortality as DV.

**Table 4:** Principal and partial effects of the independent variables (mediator) on the dependent variable (final): exitus.

**Principal factor**	**df**	**F**	***p < ***	**np^**2**^**	Power^*^
**Personal factors**					
YEAR	6	5.723	0.000	0.000	0.998^*^
OBESITY	1	4.239	0.040	0.000	0.539^*^
EPILEPSY	1	11.077	0.001	0.000	0.914^*^
**Contextual factors**					
YEAR	4	2.340	0.053	0.000	0.683^*^
UNIT	1	8.397	0.004	0.000	0.826^*^
**Interaction factors**					
EPILEPSY ^*^ YEAR	6	1.974	0.066	0.000	0.731^*^
UNIT ^*^ YEAR	6	2.645	0.014	0.000	0.866^*^
OBESITY ^*^ EPILEPSY	1	5.886	0.015	0.000	0.679^*^
OBESITY ^*^ UNIT	1	6.059	0.014	0.000	0.692^*^
EPILEPSY ^*^ YEAR	4	2.404	0.047	0.000	0.696^*^
EPILEPSY ^*^ UNIT	1	5.374	0.020	0.000	0.640^*^
AGE ^*^ EPILEPSY ^*^ YEAR	24	1.757	0.012	0.000	0.989^*^
AGE ^*^ EPILEPSY ^*^ UNIT	6	2.713	0.012	0.000	0.876^*^
OBESITY ^*^ EPILEPSY ^*^ UNIT	1	5.923	0.015	0.000	0.682^*^
OBESITY ^*^ YEAR ^*^					
UNIT	18	1.796	0.020	0.000	0.968^*^

*General linear model*.

* *Observed power of effect (only statistically significant). Full table provided in the Complimentary / Supplementary Material*.

By observing the pathologies coded for each hospital stay, we detected a significant main effect from multiple intermediate (or mediating) variables on mortality. This effect was shown for arrhythmias, dyslipidemia, and hypertension; however, the most powerful interaction in determining exitus was the joint effect of the interaction (Dyslipidemia^*^Hypertension) (Table 5).

**Table 5:** Effects of the individual process variables (ARRHYTHMIAS, DYSLIPIDEMIA, and HTN) and of context process variables (YEAR, STROKE UNITS) on the outcome variable (EXITUS).

**Principal factor**	**df**	**F (Pillais)**	***p < ***	**np^**2**^**	**power**
**Individual**					
ARRHYTHMIAS	1	1056.446	0.000	0.006	1.00^*^
DYSLIPIDEMIA	1	695.140	0.000	0.004	1.00^*^
HTN	1	18.429	0.000	0.000	0.990^*^
ARRHYTHMIAS ^*^ DYSLIPIDEMIA	1	6.748	0.009	0.000	0.738^*^
ARRHYTHMIAS ^*^ HTN	1	6.987	0.008	0.000	0.753^*^
DYSLIPIDEMIA ^*^ HTN	1	2.656	0.000	0.000	1.00^*^
**Contextual**					
STAY	3	13.534	0.000	0.000	1.00^*^
DIAGNOSES	3	4.075	0.007	0.000	0.848^*^
PROCEDURES	4	24.366	0.000	0.001	1.00^*^
READMISSION	1	12.411	0.000	0.000	0.941^*^
STAY ^*^ PROCEDURES	9	2.562	0.006	0.000	0.945^*^
STAY ^*^ PROCEDURES	12	3.691	0.000	0.000	0.999^*^
STAY ^*^ READMISSION	3	6.730	0.000	0.000	0.976^*^
DIAGNOSES ^*^ PROCEDURES	12	4.511	0.000	0.000	1.00^*^
DIAGNOSES ^*^ READMISSION	3	4.752	0.003	0.000	0.902^*^
PROCEDURES ^*^ READMISSION	4	3.088	0.015	0.000	0.815^*^
STAY ^*^ DIAGNOSES ^*^ PROCEDURES	33	2.180	0.000	0.000	1.00^*^
STAY ^*^ DIAGNOSES ^*^ READMISSION	9	2.804	0.003	0.000	0.964^*^
STAY ^*^ PROCEDURES ^*^ READMISSION	12	1.804	0.042	0.000	0.897^*^
DIAGNOSES ^*^ PROCEDUR ^*^ READMISSION	9	4.248	0.000	0.000	0.998^*^
STAY ^*^ DIAGNOSES ^*^ PROCEDURES					
^*^ READMISSION	23	1.797	0.011	0.000	0.989^*^

*Lineal General Model*.

* *Observed power (significant); HTN, Arterial Hypertension. Full table provided in the Complimentary / Supplementary Material*.

Regarding the discrete contextual variables analyzed, those with the greatest effect were length of stay and NPR, along with readmissions. However, the variables with the greatest explanatory power were the interactions of (Length of Stay^*^NDX^*^NPR), (NDX^*^NPR^*^Readmissions), (Length of Stay^*^NDX^*^Readmissions), and (Length of Stay^*^NDX^*^NPR^*^Readmissions).

### Linear Relations of Structural Prediction

The results of structural analysis or pathway analysis (SEM) showed an acceptable model of relationships. The relationship parameters of both models are presented below (Table 6).

**Table 6:** Models of structural linear results of the variables.

**Model**	**Degrees of Freedom**	**Chi-square**	***p*<**	**NFI**	**RFI**	**IFI**	**TLI**	**CFI**	**RMSEA**	**Hoelter**
										0.05–0.01
1.14 F	(119-64): 55	77103.176	0.001	0.374	0.391	0.374	0.391	0.374	0.087	178–199
2.14 F	(119-73): 46	32527.569	0.001	0.736	0.397	0.736	0.397	0.736	0.062	360–408
3.14 F	(119-82): 37	4721.698	0.001	0.963	0.935	0.963	0.926	0.963	0.026	2059–2363

#### Standardized Direct Effects

In the case of the personal variables, the predictive linear model establishes that the variable GENDER was predicted by AGE (0.259). OBESITY was negatively predicted by AGE (-0.111) and positively by GENDER (0.078). EPILEPSY was positively predicted by GENDER (0.008), and UNIT was positively predicted by AGE (0.013).

The variable ARRHYTHMIAS was significantly predicted by AGE (0.180), GENDER (0.067), OBESITY (−0.055), the number of DIAGNOSES (0.354), and PROCEDURES (−0.029). DYSLIPIDEMIA was predicted by AGE (−0.074), OBESITY (-0.064), and by EPILEPSY (−0.013). HTN was predicted by AGE (0.230), GENDER (0.041), OBESITY (0.137), EPILEPSY (0.021), and ARRHYTHMIAS (−0.103).

As for the contextual variables, the variable UNIT was significantly predicted by AGE (0.013), STAY was predicted positively by EPILEPSY (0.035) and negatively by DIAGNOSES (−0.452). The variable DIAGNOSES was predicted by AGE (0.138), EPILEPSY (0.077), YEAR (0.164), UNIT (0.107), ARRHYTHMIAS (−0.114), DYSLIPIDEMIA (0.181), HTN (0.554), and STAY (0.148). The PROCEDURES variable was predicted by AGE (−218), by STAY (0.157), and by DIAGNOSES (0.211).

Finally, the variable READMISSION was predicted negatively by DYSLIPIDEMIA (−0.027), HTN (−0.058) and PROCEDURES (−0.058), and positively by DIAGNOSES (0.102). The variable EXITUS (DEATH) was positively predicted by AGE (0.141), ARRHYTHMIAS (0.064), and READMISSIONS (0.052), and negatively predicted by DISLIPIDEMIA (−0.046), HTN (−0.046), and PROCEDURES (−0.065). All error variances were significant (*p* < 0.001). Table 7 shows the direct effects of the variables included in the model.

**Table 7:** Standardized direct effects (Default model).

	**AGE**	**GEND**	**OBES**	**EPILEP**	**YEAR**	**UNIT**	**ARR**	**DYSL**	**HTN**	**STAY**	**NDX**	**NPR**	**READM**
GENDER	0.259												
OBESIT	−0.111	0.078											
EPILEP		0.008		0.077									
ARR	0.180	0.067	−0.055								0.354^*^	−0.029	
DYSLIP	−0.074		0.064	−0.013									
HTN	0.230	0.041	0.137	0.021			−0.103						
YEAR													
UNIT	0.013												
STAY				0.035							−0.452^*^		
DIAGN	0.138				0.164	0.107	−0.114	0.181	0.554^*^	0.148			
PROC	−0.218									0.157	0.211		
READM								−0.027	−0.029		0.102	−0.058	
EXITUS	0.141						0.064	−0.046	−0.040			−0.065.	0.052

*AGE, Age; GEND, Gender; OBES, Obesity; EPILEP, Epilepsy; YEAR, Year; UNIT, Stroke Unit; ARR, arrhythmias; DYSL, Dyslipidemia; HTN, Arterial Hypertension: STAY, Hospital Stay; NDX, Number of Diagnoses; NPR, Number of Procedures; READM, Readmission*.

* *IMPORTANT EFFECT*.

#### Standardized Indirect Effects

The model also revealed multiple indirect predictions among the variables. With respect to personal variables, the predictive linear model establishes that AGE was a positive, significant, indirect predictor of OBESITY (0.020). The variable EPILEPSY was not predicted indirectly by any other variable.

The variable ARRHYTHMIA was indirectly predicted, in a positive sense, by AGE (0.080), OBESITY (0.029), EPILEPSY (0.024), UNITS (0.034), DYSLIPIDEMIA (0.088), HTN (0.145), and STAY (0.044), and in a negative sense by ARRHYTHMIAS (−0.088) and DIAGNOSES (−0.092).

DYSLIPIDEMIA was indirectly predicted, in a positive sense, by OBESITY (0.004), UNITS (0.005), ARRHYTHMIAS (0.008), and PROCEDURES (0.004), while negatively predicted by AGE (−0.042), GENDER (−0.004), EPILEPSY (−0.003), YEAR (-0.005), HTN (−0.020), STAY (−0.006), and DIAGNOSES (−0.036).

Hypertension was indirectly and negatively predicted by AGE (−0.125), GENDER (−0.003), OBESITY (−0.016), EPILEPSY (−0.036), YEAR (−0.062), UNITS, ARRHYTHMIAS (−0.044), DYSLIPIDEMIA (−0.127), HTN (−0.210), STAY (−0.070), while predicted positively by DIAGNOSES (0.073) and PROCEDURES (0.003).

In the case of contextual variables, the existence of a stroke unit (UNIT) was not indirectly predicted by any other variable in the model. Length of stay (STAY) was indirectly predicted, in a positive sense, by AGE (0.003), GENDER (0.001), DYSLIPIDEMIA (0.001), and HYPERTENSION (0.001), while negatively predicted by ARRHYTHMIAS (−0.001).

The variable DIAGNOSES was positively predicted by AGE (0.009), GENDER (0.013), OBESITY (0.082) and DYSLIPIDEMIA (0.071), and negatively predicted by EPILEPSY (−0.009), ARRHYTHMIAS (−0.048), HYPERTENSION (−0.136), DIAGNOSES (−0.246), and YEAR (−0.048).

PROCEDURES were positively predicted by AGE (0.003), GENDER (0.003), OBESITY (0.012), EPILEPSY (0.020), DYSLIPIDEMIA (0.053), HTN (0.088), YEAR (0.015) and UNITS (0.032); and negatively by ARRYHTHMIAS (−0.033) and DIAGNOSES (−0.052).

The outcome variable READMISSION was indirectly and positively predicted by the personal variables AGE (0.003), GENDER (0.002), OBESITY (0.001), EPILEPSY (0.007), DYSLIPIDEMIA (0.048), and HTN (0.048), and positively predicted by the contextual variables YEAR (0.012) and UNIT (0.003), while predicted negatively by contextual variables DIAGNOSES (−0.015) and PROCEDURES (−0.001).

The other variable, EXITUS (DEATH), was predicted positively by AGE (0.031), GENDER (0.003), HTN (0.014), DIAGNOSES (0.025), and negatively by OBESITY (−0.002), EPILEPSY (−0.002), DYSLIPIDEMIA (−0.004), UNITS (0.013), and PROCEDURES (−0.005) (see Table 8).

**Table 8:** Standardized indirect effects (Default model).

	**AGE**	**GEND**	**OBES**	**EPILEP**	**YEAR**	**UNIT**	**ARR**	**DYSL**	**HTN**	**STAY**	**NDX**	**NPR**	**READM**
GENDER													
OBESIT	0.020												
EPILEP													
ARR	0.080		0.029	0.024		0.034	−0.057	0.088	0.145^*^	0.044	−0.092		
DYSLIP	−0.042	−0.004	0.004	−0.003	−0.005	0.005	0.008	−0.012	−0.020	−0.066	−0.036	0.004	
HTN	−0.125	−0.003	−0.016	−0.036	−0.062	−0.051	−0.044	−0.127	−0.210^*^	−0.070	0.073	0.003	
YEAR													
UNIT													
STAY	0.003	0.001					−0.001	0.001	0.001				
NDX	0.009	0.013	0.082	−0.009	−0.048	0.001	−0.048	0.071	−0.136	−0.009	−0.246	0.001	
NPR	0.033	0.003	0.012	0.020	0.015	0.032	−0.033	0.053	0.088	0.029	−0.052		
READM	0.033	0.002	0.001	0.007	0.012	0.003	−0.011	0.021	0.048	0.005	−0.015	−0.001	
EXITUS	0.031	0.003	−0.011	−0.002	0.009	−0.013	0.007	−0.004	0.014	−0.009	0.025	−0.005	

*AGE, Age; GEND, Gender; OBES, Obesity; EPILEP, Epilepsy; YEAR, Year; UNIT, Stroke Unit; ARR, arrhythmias; DYSL, Dyslipidemia; HTN, Arterial Hypertension: STAY, Hospital Stay; NDX, Number of Diagnoses; NPR, Number of Procedures; READM, Readmission*.

* *IMPORTANT EFFECT*.

##### Graphic representation of the structural model

**Figure 1:**
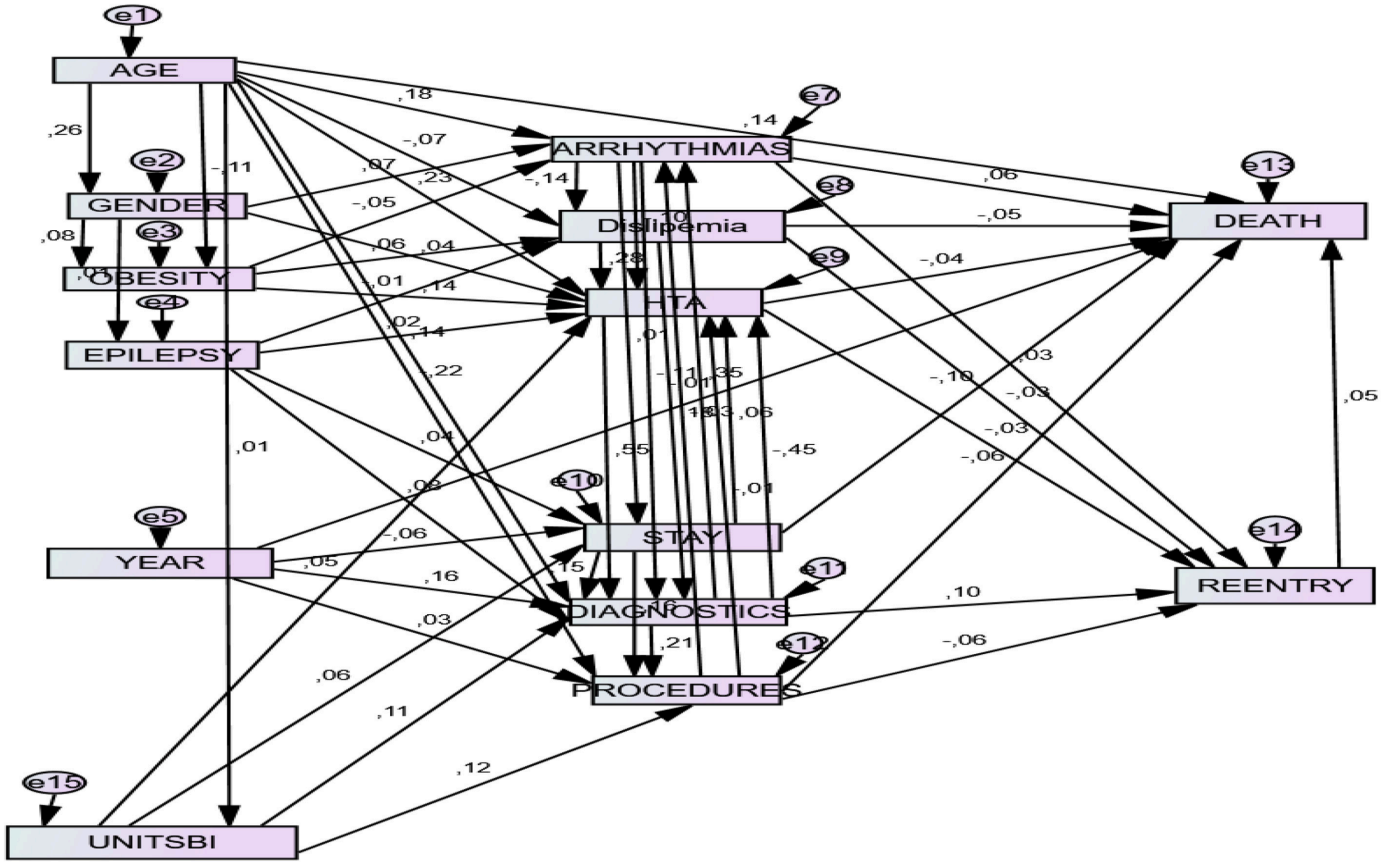
Final structural model. Unitsbi, existence of stroke unit; HTA, Hypertension; Stay, Length of stay; Reentry: readmission within 30 days under the same DRG; Diagnostics: Number of diagnoses upon discharge. Procedures: Number of procedures upon discharge.

The final model is graphically represented in Figure 1.

## Discussion

### Empirical Evidence

This investigation began with the hypothesis that each variable defined in the linear model, whether individual or contextual, would have a statistically significant effect on the intermediate variables of the established model, at the individual level and at the contextual level. These in turn would have a significant effect on the two dependent outcome variables, namely, readmissions and mortality. This hypothesis was in large measure confirmed, having verified in our SEM model that the *individual variables* made a differential, statistically significant impact on the *intermediate* (mediating) variables, and these in turn on exitus. This is not an every-variable-to-every-variable relationship; the particular predictions are made explicit below, as well as some paradoxical relationships that deserve a detailed explanation. The inferential results presented here show effects from combined variables, similar to what has been reported with prior evidence. The clearest effects were produced by the combination of multiple variables.

#### Individual Variables as Predictors

As seen in other studies, different variables were found to be statistically significant predictors of the presence of arrhythmias as comorbidity in this group of patients. In this context, arrhythmias were significantly, positively predicted by age (21, 22), obesity (23, 24) and the presence of epilepsy among the secondary diagnoses. Some of these linear associations were known previously, but had not been demonstrated to date using a predictive structural model. The literature reflects an association between epilepsy and arrhythmias, whether direct or mediated by antiepileptic treatment (25–27). In the same way, age was associated with the presence of dyslipidemia (28) and HTN (29). The association found between epilepsy and dyslipidemia is consistent with the known effect on lipids from treatment with certain anti-epileptic drugs (30, 31).

One paradoxical result is the negative prediction of dyslipidemia as a function of *age*. A possible explanation would be that stroke-affected patients suffer from vasculopathy and often arteriopathy; they are affected by different types of pathologies that are treated fundamentally with statins. Prior research has demonstrated that the use of statins increases with age. Thus, age might be negatively associated with dyslipidemia through the use of this pharmacological group in the type of patient most prevalent in this study: older people with a background of cardiovascular pathology.

On the other hand, gender (being a woman) positively predicts arrhythmias and HTN, but not dyslipidemia. The association between the female gender and the existence of certain types of arrhythmias is well-documented (32), and probably accounts for our findings. However, the limitations of our information source (CMBD) do not allow us to identify the subtypes of arrhythmias prevalent in our study sample. As for HTN, it is known to be more prevalent and more associated with men at younger ages than women, but in the situation that concerns us, several elements might explain an association with the female gender. On one hand we are working with patients affected by an ischemic stroke and not the general population; on the other hand, the more senile sectors, with higher prevalence of HTN, are also mostly female in our sample and in the general population, due to the longer life expectancy of women.

Another noteworthy result is the positive predictive role of epilepsy with respect to HTN. According to the established literature, HTN is an obvious, crucial risk factor for ischemic stroke, in the same way that stroke itself is a risk factor for developing epileptic crises. We may then suppose, in full agreement with other authors (33), that the relationship between HTN—especially if not properly controlled—and epilepsy can also develop directly, that is, even prior to development of an ischemic event.

These results concur with prior medical evidence showing that *age* positively predicts arrhythmias (21, 23) as well as HTN (29). Although the evidence is not as clear, age influences hemodynamic regulatory mechanisms, which in turn have consequences in blood pressure and brain self-regulation (29). A paradoxical result is the negative prediction of dyslipidemia.

*Obesity*, for its part, negatively predicts arrhythmias, but positively predicts dyslipidemia and HTN. A consistent model of obesity as a positive predictor of dyslipidemia and HTN is evident and well-documented (34, 35); this falls in line with the relatively new concept of obesity as a chronic, recurring, progressive disease, as suggested by Bray et al. (36). Finally, in our understanding to date, there seems to be no clear association of obesity and arrhythmias, or at the least, it would occur through some mechanism not yet understood.

There are substantial associations between variables of individual characteristics and the two main dependent variables (readmissions and exitus); in a few cases, they seem paradoxical or difficult to explain, thus indicating a need to investigate some of the predictive effects that were found. Both dyslipidemia and NPR are significant, negative predictors of readmissions, while NDX is a significant, positive predictor. Increased procedural effort or therapeutic intensity can explain the direction of the NPR-Readmissions prediction, such that where greater effort is applied, there is less likelihood of being readmitted to hospital for the same reason in the 30 days following discharge. Similarly, when patients have a greater number of diagnoses (greater comorbidity), prediction of readmissions is positive, demonstrating that the patient's overall complexity undoubtedly influences his or her prognosis.

Elsewhere, the evidence showed dyslipidemia and NPR as negative predictors of mortality, while age and the existence of arrhythmias were positive predictors. It seems logical that more elderly patients, and patients affected by arrhythmias (also more frequent at advanced ages), would have greater mortality. The negative association between dyslipidemia and mortality, to our understanding, can only be understood in that dyslipidemic patients receive greater procedural effort, and probably undergo more frequent medical checks. This assertion is supported by the direct, significant, negative prediction that occurs between the number of procedures applied, and mortality.

To complete this section, we must make note of the central, core prediction between the two dependent variables. Just as each different individual variable on its own has been related through different mechanisms to each of the dependent variables, there is an obvious, significant, and very powerful prediction between readmissions and mortality. This association has been cited in many studies on a variety of pathologies, and we believe it lends even greater biological plausibility to the structural model (37–39).

#### Contextual Variables as Predictors

There were also statistically significant effects from the *contextual variables*. *Year* was confirmed to have a negative effect on length of stay and on in-hospital mortality. The effect on mortality was mediated by NDX and NPR, variables that in turn depend directly on the existence of stroke units and the ongoing creation of such units during the study period. The period analyzed in this study was a time of marked change, where improved stroke care, both in therapeutic terms and in organization of care, prompted a drop in average length of stay and in short-term mortality—and consequently in in-hospital mortality, which we are analyzing here (16, 40).

In this context, where there is higher patient comorbidity (with NDX as the proxy variable for comorbidity), there are higher levels of 30-day readmissions, and secondarily, there are the above-mentioned increases in mortality. As for NPR, considered a proxy variable for the degree of therapeutic effort applied to the patient, we find that with greater effort, there is a decrease in readmissions and in mortality. Both variables are closely related to the existence of stroke units, such that procedural effort is objectively greater within these units than in conventional hospitalization (41).

Although the moment in time (Year) predicted shorter hospital stays, within stroke units there was greater likelihood of longer stays throughout the whole study period. The most important effect found was that the existence of *stroke units* positively predicted length of STAY, as commented. These units admit the more complex patients in particular (greater comorbidity or NDX), and apply greater therapeutic and procedural effort (higher NPR), which would explain the decrease in both mortality and in readmissions; according to other authors, however, a paradoxical effect can occur due to the patient's own complexity (42, 43). The contextual variable that most clearly affects decreased mortality is procedural effort (NPR), which in turn is higher in stroke units and in patients with greater complexity (NDX); both of these variables (diagnoses and procedures) are associated with the stroke units themselves, due to the type of cases that are admitted in these units (44).

Another noteworthy result is the predictive effect of individual mediating variables on context variables. The most interesting result, from the point of view of how the healthcare system affects disease in subjects, is that the number of DIAGNOSES negatively predicts length of STAY, but positively predicts an increased number of PROCEDURES. This may be interpreted as more complex patients having shorter hospital stays because of the high levels of accumulated mortality in this group. The patient's diagnostic complexity (NDX) itself would lead to greater procedural effort (NPR), but there may also be mechanisms that limit therapeutic effort at the most advanced ages (45). In any case, according to our criteria, the model has the capacity to explain these complex associations that are made evident through structural models and that underlie the clinician's thinking and the physiopathology of disease in a stroke.

## Clinical Implications

Regarding the importance of the proposed *illustrated algorithm*, the present analysis yields an empirical model that incorporates a macro and micro view of predictive relationships between the independent, mediating, and outcome factors of the subjects' health in interaction with the contextual, organizational factors. In our view, this model has unquestionable epidemiological value for revealing probabilistic predictive relationships between personal and contextual factors, thereby enabling healthcare organizations to understand and make decisions regarding the detection of diseases that bring increased likelihood of others. It also enables large-scale assessment of the adequacy of resources deployed as a function of the pathologies analyzed, opening the way to cost-benefit analyses. Some previous analyses have contributed evidence in this line of work, using different methodologies (8, 46).

The results of this study are also relevant from the point of view of *clinical management*. Attention to the value of contextual elements (mainly managerial and organizational elements like stroke units) would unquestionably contribute to improved clinical care for the patient and to organizational efficiency itself. An understanding of how the individual and contextual elements of stroke are related to each other gives us a broad, ambitious view of this scenario, now supported by a structural model that provides empirical evidence, in contrast to the formerly fragmented or non-existent evidence in prior contributions to our understanding of this disease.

## Methodological Contributions

Contributions from this type of analysis of *large clinical-administrative databases* are obvious. First, this approach goes beyond the classic, correlational methodology that establishes covariation relationships between study variables but has many limitations with respect to establishing causal relationships. In fact, certain prior studies have shown that when empirical models are based on associations between variables, and an SEM model is later developed, some of the previous association relations are not sustained in the new structural model, because of accumulated measurement errors. Second, while carrying out prior inferential analyses ensures that interdependence (or causal) relationships between variables are consistent, this type of analysis is unable to present such relationships in a combined, multidirectional manner, but only as limited to each multivariate analysis. Third, the SEM model makes it possible to establish structural multi-directionality of causation through path analysis. Consequently, this type of analysis would be appropriate to an R&D&I Department (47) within the hospital context, where it would be possible to test the efficiency of hospital interventions and healthcare resources (48, 49).

## Limitations and Prospects

A first methodological limitation is that no latent variables have been defined in the model. Latent variables can establish a generic relation between constructs, but not the specific ones that we wanted to find. In our case, we have tried to define the causal relationships between observable variables. From our point of view, this precise relationship is very important.

The data are taken from non-lysed strokes. The clinical situation today is a different one (intravenous fibrinolysis and mechanical thrombectomy), where the role of the stroke unit is even more critical. However, given the high prevalence of this subtype of stroke (ischemic and not subject to reperfusion), we think that establishing a predictive empirical model with personal and hospital variables is of great relevance. It would be interesting to replicate the study with patients who have received treatment for acute stroke, when enough data become available. We believe that the future inclusion of patients subjected to mechanical or chemical reperfusion would probably modify the outputs in the sense of less sequential morbidity, decreased length of stay, and lower mortality in stroke units and even in general hospitalization. We also consider that the contextual dependent variable “readmissions” would be favorably diminished by the inclusion of these new therapeutic techniques. Even in the case of non-lysed stroke, this replication in a real cohort would make it possible to simplify and further divide up the elements of the final model. We could learn more precisely which elements might be implemented in routine clinical care in order to optimize outcomes.

Working with these massive clinical-administrative databases has the advantage of the great statistical power of a large sample size, but such databases are not free from significant drawbacks. On one hand, the data reflect the in-hospital situation exclusively, possibly leading to an external validity issue; in our particular case, acute stroke patients are rarely addressed on an outpatient basis, so we consider this bias to be minimal. We also must consider that the information is limited by the quality of the diagnostic and procedural codings themselves, and that this quality is rather uneven, not only geographically (different healthcare regions) but also over time (the study period), fortunately the latter tends toward improvement.

In addition, we must consider the limitation that variables such as “epilepsy” imply, where we cannot identify whether it is occurring as a result of the stroke or whether the patient has suffered this pathology for some time. This obvious database limitation in not differentiating certain secondary diagnoses as complications or as comorbidity is only partially compensated by the high sample size and the diagnostic position: epilepsy encoded in the second diagnostic position is understood to be an acute complication, while in lower positions it is more likely to be a preexisting comorbidity.

Finally, we must take into account the very critical patients who die shortly after admission: their chronic pathologies are often under recorded, possibly distorting the statistical results and even provoking paradoxical results. The well-known Jencks bias, a phenomenon described in Jencks et al. (50), has been confirmed in multiple studies. Studies by Dahlin et al. (51) are most noteworthy, where under recording was proven to be a constant, even when as many as 25 diagnoses had been reported upon discharge. For all these reasons, such biases in the information source are difficult to control, but given the sample size, power and level of detail, this source provides extremely valuable information for patient care and for improved organizational management.

## Author Contributions

JdlF development of the conceptual idea. Statistical methodology Global drafting of the manuscript. JG-T general review of the manuscript. Introduction and review-writing of the discussion. MI-E general review of the manuscript and partial wording of the discussion. GS global review of the manuscript. Statistical review. AG-U review of the design evaluation process used. JF-P review of the global English level of the manuscript.

## Conflict of Interest Statement

The authors declare that the research was conducted in the absence of any commercial or financial relationships that could be construed as a potential conflict of interest.
