# Supplementary material for: Structural Equation Model (SEM) of Stroke Mortality in Spanish Inpatient Hospital Settings: The Role of Individual and Contextual Factors
Source: Front Neurol. 2019 May 17;10:498. doi: 10.3389/fneur.2019.00498 (PMC6533919; doi:10.3389/fneur.2019.00498)
Supplement: Supplementary file 1 [file Data_Sheet_1.pdf]

COMPLEMENTARY MATERIAL

Table 2.
Principal and partial effects of independent variables (presage) on dependent variables (process). General Linear model. Full Table

Main effect	Value	F (Pillai)	df	g error	*p*<.	np²	Not centr.	Power
AGE_r	.002	7.748	42.000	1061436.000	.000	.000	325.423	1.00*
GENDER	.000	.578b	7.000	176901.000	.775	.000	4.046	.255
OBESITY	.001	13.291b	7.000	176901.000	.000	.001	93.036	1.00*
EPILEPS	.000	7.785b	7.000	176901.000	.000	.000	54.492	1.00*
YEAR	.000	2.009	28.000	707616.000	.001	.000	56.243	.999*
UNIT	.000	9.428b	7.000	176901.000	.000	.000	65.993	1.00*

Partial effect (only significant partial effects)

IV	DV	Value	df	g Error	F (Pillai)	*p*<.	np²	Not centr.	Power
GENDER	ARRHYTHMIAS	,201	1	,201	1,069	,301	,000	1,069	,179
	DYSLIPIDEMIA	,071	1	,071	,320	,572	,000	,320	,087
	HTN	,215	1	,215	,979	,322	,000	,979	,168
	STAY	,261	1	,261	,013	,910	,000	,013	,051
	DIAGNOSES	3,879	1	3,879	,498	,481	,000	,498	,109
	PROCEDURES	4,115	1	4,115	,741	,389	,000	,741	,138
	READMISSION	,014	1	,014	,304	,581	,000	,304	,086
AGE_r * OBESITY	ARRHYTHMIAS	,948	6	,158	,839	,540	,000	5,033	,338
	DYSLIPIDEMIA	,663	6	,110	,499	,810	,000	2,992	,205
	HTN	1,016	6	,169	,770	,593	,000	4,622	,311
	STAY	191,243	6	31,874	1,562	,154	,000	9,370	,610
	DIAGNOSES	6,237	6	1,040	,133	,992	,000	,800	,084
	PROCEDURES	18,344	6	3,057	,551	,770	,000	3,304	,225
	READMISSION	,056	6	,009	,205	,975	,000	1,229	,105
AGE_r * EPILEPSY	ARRHYTHMIAS	,275	6	,046	,243	,962	,000	1,458	,117
	DYSLIPIDEMIA	436	6	,239	1,080	,371	,000	6,482	,435
	HTN	,617	6	,103	,468	,833	,000	2,807	,194
	STAY	114,666	6	19,111	,936	,467	,000	5,618	,377
	DIAGNOSES	18,126	6	3,021	,388	,887	,000	2,326	,165
	PROCEDURES	12,672	6	2,112	,380	,892	,000	2,283	,162
	READMISSION	,032	6	,005	,117	,994	,000	,703	,080
Age_r * UNITI	ARRHYTHMIAS	,606	6	,101	,536	,781	,000	3,216	,219
	DYSLIPIDEMIA	,712	6	,285	1,288	,259	,000	7,730	,514
	HTN	,558	6	,093	,423	,865	,000	2,535	,177
	STAY	91,992	6	15,332	,751	,608	,000	4,507	,303
	DIAGNOSES	56,699	6	9,450	1,213	,296	,000	7,276	,486
	PROCEDURES	35,938	6	5,990	1,079	,372	,000	6,474	,434
	READMISSION	,097	6	,016	,357	,906	,000	2,140	,154
YEAR * GENDER	ARRHYTHMIAS	,852	4	,213	1,131	,340	,000	4,522	,360
	DYSLIPIDEMIA	,673	4	,168	,760	,551	,000	3,040	,247
	HTN	,648	4	,162	,736	,567	,000	2,945	,240
	STAY	50,852	4	12,713	,623	,646	,000	2,492	,207
	DIAGNOSES	29,490	4	7,373	,946	,436	,000	3,784	,304
	PROCEDURES	51,333	4	12,833	2,312	,055	,000	9,247	,677
	READMISSION	,104	4	,026	,570	,685	,000	2,279	,191
YEAR * EPILEPSY	ARRHYTHMIAS	,783	4	,196	1,039	,385	,000	4,156	,332
	DYSLIPIDEMIA	,221	4	,055	,249	,910	,000	,997	,105
	HTN	,493	4	,123	,560	,692	,000	2,240	,188
	STAY	143,213	4	35,803	1,754	,135	,000	7,017	,541

	DIAGNOSES	9,574	4	2,393	,307	,873	,000	1,229	,120
	PROCEDURES	4,195	4	1,049	,189	,944	,000	,756	,091
	READMISSION	,156	4	,039	,854	,491	,000	3,416	,276
YEAR * UNITS	ARRHYTHMIAS	,346	4	,087	,460	,765	,000	1,839	,160
	DYSLIPIDEMIA	,260	4	,065	,294	,882	,000	1,174	,116
	HTN	,216	4	,054	,246	,912	,000	,984	,105
	STAY	104,119	4	26,030	1,275	,277	,000	5,101	,404
	DIAGNOSES	3,464	4	,866	,111	,979	,000	,445	,073
	PROCEDURES	26,285	4	6,571	1,184	,316	,000	4,735	,376
	READMISSION	,063	4	,016	,347	,846	,000	1,389	,130
GENDER * EPILEPSY	ARRHYTHMIAS	,324	1	,324	1,718	,190	,000	1,718	,259
	DISLIPEMIA	,372	1	,372	1,678	,195	,000	1,678	,254
	HTN	,070	1	,070	,320	,572	,000	,320	,087
	STAY	5,205	1	5,205	,255	,614	,000	,255	,080
	DIAGNOSES	,449	1	,449	,058	,810	,000	,058	,057
	PROCEDURES	6,185	1	6,185	1,114	,291	,000	1,114	,184
	READMISSION	,053	1	,053	1,166	,280	,000	1,166	,191
GENDER * UNIT	ARRHYTHMIAS	,496	1	,496	2,635	,105	,000	2,635	,368
	DISLIPEMIA	,476	1	,476	2,150	,143	,000	2,150	,311
	HTN	,066	1	,066	,302	,583	,000	,302	,085
	STAY	15,225	1	15,225	,746	,388	,000	,746	,139
	DIAGNOSES	2,618	1	2,618	,336	,562	,000	,336	,089
	PROCEDURES	5,061	1	5,061	,912	,340	,000	,912	,159
	READMISSION	,034	1	,034	,743	,389	,000	,743	,138
OBESITY * EPILEP	ARRHYTHMIAS	,011	1	,011	,060	,806	,000	,060	,057
	DYSLIPIDEMIA	,011	1	,011	,048	,827	,000	,048	,055
	HTN	,168	1	,168	,764	,382	,000	,764	,141
	STAY	30,887	1	30,887	1,513	,219	,000	1,513	,233
	DIAGNOSES	,026	1	,026	,003	,954	,000	,003	,050
	PROCEDURES	2,290	1	2,290	,412	,521	,000	,412	,098
	READMISSION	,044	1	,044	,961	,327	,000	,961	,165
OBESITY * UNIT	ARRHYTHMIAS	,006	1	,006	,032	,859	,000	,032	,054
	DYSLIPIDEMIA	,025	1	,025	,115	,735	,000	,115	,063
	HTN	,001	1	,001	,002	,962	,000	,002	,050
	STAY	26,018	1	26,018	1,275	,259	,000	1,275	,204
	DIAGNOSES	,392	1	,392	,050	,823	,000	,050	,056
	PROCEDURES	,606	1	,606	,109	,741	,000	,109	,063
	READMISSION	,019	1	,019	,426	,514	,000	,426	,100
Age_r * YEAR * GENDER	ARRHYTHMIAS	3,821	24	,159	,845	,680	,000	20,287	,743
	DYSLIPIDEMIA	5,292	24	,220	,995	,468	,000	23,888	,831
	HTN	5,473	24	,228	1,037	,412	,000	24,886	,851
	STAY	541,904	24	22,579	1,106	,326	,000	26,551	,880
	DIAGNOSES	119,041	24	4,960	,636	,912	,000	15,275	,578
	PROCEDURES	134,815	24	5,617	1,012	,445	,000	24,285	,839
	READMISSION	,946	24	,039	,865	,652	,000	20,771	,757
Age_r * YEAR * EPILEPSY	ARRHYTHMIAS	2,376	24	,099	,526	,972	,000	12,613	,474
	DYSLIPIDEMIA	6,277	24	,262	1,181	,246	,000	28,335	,905
	HTN	5,088	24	,212	,964	,512	,000	23,138	,815
	STAY	571,748	24	23,823	1,167	,260	,000	28,013	,901
	DIAGNOSES	90,750	24	3,781	,485	,984	,000	11,645	,435
	PROCEDURES	178,267	24	7,428	1,338	,124	,000	32,112	,945
	READMISSION	1,161	24	,048	1,062	,379	,000	25,492	,862
Age_r * YEAR * UNITSBI	ARRHYTHMIAS	4,167	24	,174	,922	,572	,000	22,123	,791
	DYSLIPIDEMIA	4,507	24	,188	,848	,677	,000	20,343	,745
	HTN	2,408	24	,100	,456	,989	,000	10,949	,406

	STAY	466,207	24	19,425	,952	,529	,000	22,842	,808
	DIAGNOSES	85,697	24	3,571	,458	,989	,000	10,997	,408
	PROCEDURES	179,287	24	7,470	1,346	,120	,000	32,296	,946
	READMISSION	,885	24	,037	,809	,729	,000	19,427	,718
Age_r * GENDER * OBESITY	ARRHYTHMIAS	,256	6	,043	,226	,968	,000	1,358	,111
	DYSLIPIDEMIA	1,618	6	,270	1,217	,294	,000	7,304	,487
	HTN	1,191	6	,198	,902	,492	,000	5,414	,364
	STAY	126,137	6	21,023	1,030	,403	,000	6,180	,415
	DIAGNOSES	14,450	6	2,408	,309	,933	,000	1,854	,138
	PROCEDURES	16,469	6	2,745	,494	,813	,000	2,967	,204
	READMISSION	,175	6	,029	,640	,698	,000	3,840	,259
Age_r * GENDER * EPILEPSY	ARRHYTHMIAS	,493	6	,082	,436	,855	,000	2,615	,182
	DYSLIPIDEMIA	,413	6	,069	,311	,932	,000	1,866	,139
	HTN	1,245	6	,208	,944	,462	,000	5,663	,380
	STAY	130,643	6	21,774	1,067	,380	,000	6,401	,429
	DIAGNOSES	35,246	6	5,874	,754	,606	,000	4,523	,304
	PROCEDURES	32,004	6	5,334	,961	,450	,000	5,765	,387
	READMISSION	,326	6	,054	1,195	,305	,000	7,170	,479
Age_r * GENDER * UNITSBI	ARRHYTHMIAS	,752	6	,125	,665	,678	,000	3,991	,269
	DYSLIPIDEMIA	,671	6	,112	,505	,805	,000	3,028	,207
	HTN	1,221	6	,204	,925	,475	,000	5,552	,373
	STAY	168,048	6	28,008	1,372	,221	,000	8,234	,545
	DIAGNOSES	32,174	6	5,362	,688	,659	,000	4,129	,278
	PROCEDURES	35,917	6	5,986	1,078	,373	,000	6,470	,434
	READMISSION	,263	6	,044	,963	,448	,000	5,778	,388
Age_r * OBESITY * EPILEPSY	ARRHYTHMIAS	1,117	6	,186	,988	,431	,000	5,927	,398
	DYSLIPIDEMIA	,195	6	,032	,147	,990	,000	,879	,088
	HTN	,501	6	,084	,380	,892	,000	2,279	,162
	STAY	89,717	6	14,953	,733	,623	,000	4,396	,296
	DIAGNOSES	8,189	6	1,365	,175	,984	,000	1,051	,096
	PROCEDURES	30,175	6	5,029	,906	,489	,000	5,435	,365
	READMISSION	,061	6	,010	,224	,969	,000	1,342	,111
Age_r * OBESITY * UNIT	ARRHYTHMIAS	,388	6	,065	,344	,914	,000	2,061	,150
	DYSLIPIDEMIA	1,096	6	,183	,824	,551	,000	4,946	,333
	HTN	,620	6	,103	,470	,831	,000	2,818	,194
	STAY	100,845	6	16,807	,823	,551	,000	4,941	,332
	DIAGNOSES	25,064	6	4,177	,536	,781	,000	3,216	,219
	PROCEDURES	9,075	6	1,512	,272	,950	,000	1,635	,126
	READMISSION	,069	6	,011	,251	,959	,000	1,506	,119
YEAR * OBESITY * UNIT	ARRHYTHMIAS	1,059	4	,265	1,406	,229	,000	5,624	,443
	DYSLIPIDEMIA	,695	4	,174	,784	,535	,000	3,135	,254
	HTN	,040	4	,010	,045	,996	,000	,181	,059
	STAY	141,543	4	35,386	1,734	,139	,000	6,935	,536
	DIAGNOSES	5,414	4	1,354	,174	,952	,000	,695	,087
	PROCEDURES	11,374	4	2,844	,512	,727	,000	2,049	,175
	READMISSION	,081	4	,020	,442	,778	,000	1,769	,155
YEAR * EPILEPSY * UNITSBI	ARRHYTHMIAS	,400	4	,100	,531	,713	,000	2,123	,180
	DYSLIPIDEMIA	,754	4	,189	,851	,492	,000	3,405	,275
	HTN	,129	4	,032	,146	,965	,000	,585	,081
	STAY	111,990	4	27,997	1,372	,241	,000	5,487	,433
	DIAGNOSES	9,807	4	2,452	,315	,868	,000	1,258	,122
	PROCEDURES	12,175	4	3,044	,548	,700	,000	2,193	,185

	READMISSION	,076	4	,019	,415	,798	,000	1,660	,148
GENDER * OBESITY * EPILEPSY	ARRHYTHMIAS	,583	1	,583	3,093	,079	,000	3,093	,420
	DYSLIPIDEMIA	,350	1	,350	1,581	,209	,000	1,581	,242
	HTN	,000	1	,000	,001	,972	,000	,001	,050
	STAY	,990	1	,990	,049	,826	,000	,049	,056
	DIAGNOSES	,952	1	,952	,122	,727	,000	,122	,064
	PROCEDURES	,343	1	,343	,062	,804	,000	,062	,057
	READMISSION	,023	1	,023	,505	,477	,000	,505	,110
GENDER * OBESITY * UNITSBI	ARRHYTHMIAS	,718	1	,718	3,811	,051	,000	3,811	,497*
	DYSLIPIDEMIA	,543	1	,543	2,453	,117	,000	2,453	,347
	HTN	,084	1	,084	,380	,538	,000	,380	,095
	STAY	5,111	1	5,111	,250	,617	,000	,250	,079
	DIAGNOSES	6,125	1	6,125	,786	,375	,000	,786	,144
	PROCEDURES	1,353	1	1,353	,244	,621	,000	,244	,078
	READMISSION	,010	1	,010	,209	,647	,000	,209	,074
GENDER * EPILEPSY * UNITSBI	ARRHYTHMIAS	,337	1	,337	1,789	,181	,000	1,789	,267
	DYSLIPIDEMIA	,413	1	,413	1,864	,172	,000	1,864	,276
	HTN	,081	1	,081	,366	,545	,000	,366	,093
	STAY	3,308	1	3,308	,162	,687	,000	,162	,069
	DIAGNOSES	6,068	1	6,068	,779	,378	,000	,779	,143
	PROCEDURES	7,580	1	7,580	1,365	,243	,000	1,365	,215
	READMISSION	,031	1	,031	,685	,408	,000	,685	,131
OBESITY * EPILEPSY * UNIT	ARRHYTHMIAS	,026	1	,026	,138	,711	,000	,138	,066
	DYSLIPIDEMIA	,014	1	,014	,062	,804	,000	,062	,057
	HTN	,164	1	,164	,746	,388	,000	,746	,139
	STAY	10,025	1	10,025	,491	,483	,000	,491	,108
	DIAGNOSES	,232	1	,232	,030	,863	,000	,030	,053
	PROCEDURES	6,337	1	6,337	1,142	,285	,000	1,142	,188
	READMISSION	,012	1	,012	,254	,614	,000	,254	,080
AGE_r * YEAR * GENDER * EPILEPSY	ARRHYTHMIAS	4,059	22	,185	,980	,487	,000	21,550	,795
	DYSLIPIDEMIA	5,157	22	,234	1,058	,386	,000	23,279	,835
	HTN	3,723	22	,169	,769	,767	,000	16,928	,659
	STAY	404,317	22	18,378	,900	,595	,000	19,810	,749
	DIAGNOSES	116,645	22	5,302	,680	,864	,000	14,968	,587
	PROCEDURES	134,431	22	6,111	1,101	,336	,000	24,216	,853
	READMISSION	,991	22	,045	,989	,474	,000	21,761	,800
AGE_r * YEAR * OBESITY * EPILEPSY	ARRHYTHMIAS	3,366	18	,187	,993	,464	,000	17,871	,735
	DYSLIPIDEMIA	4,109	18	,228	1,030	,420	,000	18,547	,756
	HTN	4,040	18	,224	1,021	,431	,000	18,371	,751
	STAY	356,758	18	19,820	,971	,490	,000	17,480	,723
	DIAGNOSES	98,575	18	5,476	,703	,812	,000	12,649	,540
	PROCEDURES	122,005	18	6,778	1,221	,233	,000	21,977	,843
	READMISSION	,437	18	,024	,533	,944	,000	9,600	,404
AGE_r * YEAR * OBESITY * UNIT	ARRHYTHMIAS	2,622	22	,119	,633	,904	,000	13,922	,546
	DYSLIPIDEMIA	5,526	22	,251	1,134	,300	,000	24,945	,867
	HTN	2,821	22	,128	,583	,938	,000	12,828	,502
	STAY	302,146	22	13,734	,673	,871	,000	14,804	,581
	DIAGNOSES	121,049	22	5,502	,706	,838	,000	15,533	,609
	PROCEDURES	131,651	22	5,984	1,078	,362	,000	23,715	,843

	READMISSION	,506	22	,023	,505	,973	,000	11,109	,431
Age_r * GENDER * OBESITY * EPILEPSY	ARRHYTHMIAS	,131	5	,026	,139	,983	,000	,694	,083
	DYSLIPIDEMIA	1,106	5	,221	,999	,417	,000	4,993	,362
	HTN	,813	5	,163	,739	,594	,000	3,695	,270
	STAY	139,220	5	27,844	1,364	,234	,000	6,821	,489
	DIAGNOSES	22,156	5	4,431	,569	,724	,000	2,843	,212
	PROCEDURES	12,536	5	2,507	,452	,812	,000	2,258	,173
	READMISSION	,283	5	,057	1,245	,285	,000	6,226	,448
Age_r * GENDER * OBESITY * UNIT	ARRHYTHMIAS	,612	6	,102	,541	,777	,000	3,246	,221
	DYSLIPIDEMIA	2,356	6	,393	1,772	,100	,000	10,633	,676
	HTN	,749	6	,125	,568	,756	,000	3,406	,231
	STAY	180,287	6	30,048	1,472	,183	,000	8,833	,580
	DIAGNOSES	50,494	6	8,416	1,080	,372	,000	6,479	,434
	PROCEDURES	34,403	6	5,734	1,033	,401	,000	6,197	,416
	READMISSION	,062	6	,010	,227	,968	,000	1,361	,112
EDAD_r * OBESITY * EPILEPSY * UNITSBI	ARRHYTHMIAS	,281	4	,070	,373	,828	,000	1,491	,137
	DYSLIPIDEMIA	,707	4	,177	,797	,527	,000	3,190	,258
	HTN	,395	4	,099	,449	,773	,000	1,797	,157
	STAY	26,523	4	6,631	,325	,861	,000	1,300	,124
	DIAGNOSES	26,082	4	6,521	,837	,502	,000	3,347	,270
	PROCEDURES	12,886	4	3,221	,580	,677	,000	2,321	,194
	READMISSION	,043	4	,011	,234	,919	,000	,936	,102
YEAR * GENDER * OBESITY * EPILEPSY	ARRHYTHMIAS	1,102	4	,276	1,463	,210	,000	5,853	,459
	DYSLIPIDEMIA	1,363	4	,341	1,539	,188	,000	6,154	,481
	HTN	1,453	4	,363	1,652	,158	,000	6,608	,513
	STAY	34,651	4	8,663	,424	,791	,000	1,698	,151
	DIAGNOSES	20,990	4	5,247	,673	,610	,000	2,693	,221
	PROCEDURES	41,680	4	10,420	1,877	,111	,000	7,508	,574
	READMISSION	,079	4	,020	,431	,786	,000	1,724	,152
YEAR * GENDER * OBESITY * UNITS	ARRHYTHMIAS	1,584	4	,396	2,103	,078	,000	8,410	,630
	DYSLIPIDEMIA	1,260	4	,315	1,422	,224	,000	5,689	,447
	HTN	1,007	4	,252	1,144	,333	,000	4,578	,364
	STAY	8,360	4	2,090	,102	,982	,000	,410	,071
	DIAGNOSES	12,409	4	3,102	,398	,810	,000	1,592	,144
	PROCEDURES	35,479	4	8,870	1,598	,172	,000	6,391	,498
	READMISSION	,044	4	,011	,240	,916	,000	,961	,103
YEAR * OBESITY * EPILEPSY * UNIT	ARRHYTHMIAS	1,618	4	,404	2,147	,072	,000	8,587	,640
	DYSLIPIDEMIA	1,374	4	,343	1,551	,185	,000	6,202	,485
	HTN	,337	4	,084	,383	,821	,000	1,533	,140
	STAY	170,720	4	42,680	2,091	,079	,000	8,365	,627
	DIAGNOSES	6,245	4	1,561	,200	,938	,000	,801	,094
	PROCEDURES	10,668	4	2,667	,480	,750	,000	1,922	,166
	READMISSION	,075	4	,019	,414	,799	,000	1,655	,148
GENDER * OBESITY *									

EPILEPSY * UNITSBI	ARRHYTHMIAS	,513	1	,513	2,723	,099	,000	2,723	,378
	DYSLIPIDEMIA	,598	1	,598	2,700	,100	,000	2,700	,376
	HTN	,051	1	,051	,232	,630	,000	,232	,077
	STAY	,068	1	,068	,003	,954	,000	,003	,050
	DIAGNOSES	10,996	1	10,996	1,411	,235	,000	1,411	,221
	PROCEDURES	2,898	1	2,898	,522	,470	,000	,522	,112
	READMISSION	,019	1	,019	,414	,520	,000	,414	,099
AGE_r * YEAR * GENDER * OBESITY * UNITSBI	ARRHYTHMIAS	2,785	18	,155	,821	,677	,000	14,783	,627
	DYSLIPIDEMIA	4,872	18	,271	1,222	,232	,000	21,991	,843
	HTN	3,173	18	,176	,802	,701	,000	14,430	,613
	STAY	237,465	18	13,192	,646	,866	,000	11,635	,496
	DIAGNOSES	103,051	18	5,725	,735	,778	,000	13,224	,564
	PROCEDURES	139,167	18	7,731	1,393	,123	,000	25,069	,899
	READMISSION	,958	18	,053	1,169	,277	,000	21,043	,822
Age_r * YEAR * OBESITY * EPILEPSY * UNITSBI	ARRHYTHMIAS	,064	1	,064	,340	,560	,000	,340	,090
	DYSLIPIDEMIA	,081	1	,081	,366	,545	,000	,366	,093
	HTN	,113	1	,113	,513	,474	,000	,513	,111
	STAY	23,512	1	23,512	1,152	,283	,000	1,152	,189
	DIAGNOSES	5,582	1	5,582	,716	,397	,000	,716	,135
	PROCEDURES	10,745	1	10,745	1,936	,164	,000	1,936	,285
	READMISSION	,005	1	,005	,103	,748	,000	,103	,062
Age_r * GENDER * OBESITY * EPILEPSY * UNIT	ARRHYTHMIAS	,423	1	,423	2,247	,134	,000	2,247	,323
	DYSLIPIDEMIA	,009	1	,009	,041	,840	,000	,041	,055
	HTN	,182	1	,182	,828	,363	,000	,828	,149
	STAY	67,678	1	67,678	3,316	,069	,000	3,316	,445
	DIAGNOSES	2,814	1	2,814	,361	,548	,000	,361	,092
	PROCEDURES	14,470	1	14,470	2,607	,106	,000	2,607	,365
	READMISSION	,001	1	,001	,025	,875	,000	,025	,053
Error	Corrected total								
	ARRHYTHMIAS	35909,254		177371					
	DYSLIPIDEMIA	40722,551		177371					
	HTN	40568,067		177371					
	STAY	3653452,478		177371					
	DIAGNOSES	1525014,544		177371					
	PROCEDURES	1054394,117		177371					
	READMISSION	8084,526		177371					

a R-squared = ,072 (adjusted R-squared = ,070)
b R-squared = ,038 (Adjusted R-squared= ,035)
c R-squared = ,041 (Adjusted R-squared= ,039)
d R-squared = ,012 (Adjusted R-squared= ,009)
e R-squared = ,096 (Adjusted R-squared= ,094)
f R-squared = ,069 (Adjusted R-squared= ,066)
g R-squared = ,004 (Adjusted R-squared= ,001)
h Calculated using alpha = ,05

Note: * Observed power of effect (only statistically significant)

Table 4.
Principal and partial effects of the independent variables (mediator) on the dependent variable (final): exitus. General linear model. Full Table

IVs	*Type III*	*df*	*Quadr*	*F*	*p<*	*np²*	*not centr.*	*Power**
Principal factor	Type III	df	Quadr	F	*p<*	np²	not centr.	Power*
Personal factors								
YEAR GP.	2,116	6	,353	5,723	,000	,000	34,339	,998*
GENDER	,008	1	,008	,138	,710	,000	,138	,066
OBESITY	,261	1	,261	4,239	,040	,000	4,239	,539*
EPILEPSY	,683	1	,683	11,077	,001	,000	11,077	,914*
Contextual factors								
YEAR	,577	4	,144	2,340	,053	,000	9,362	,683*
UNIT	,517	1	,517	8,397	,004	,000	8,397	,826*
Interaction factors								
GENDER * OBESITY	,126	1	,126	2,039	,153	,000	2,039	,298
GENDER * EPILEPSY	,078	1	,078	1,262	,261	,000	1,262	,202
GENDER * YEAR	,151	4	,038	,614	,652	,000	2,457	,204
GENDER * UNITSBI	,054	1	,054	,878	,349	,000	,878	,155
GENDER * YEAR GP.	,074	6	,012	,199	,977	,000	1,193	,103
OBESITY * YEAR GP.	,672	6	,112	1,817	,092	,000	10,900	,689
EPILEPSY * YEAR GP.	,730	6	,122	1,974	,066	,000	11,843	,731*
UNIT * YEAR GP.	,978	6	,163	2,645	,014	,000	15,873	,866*
OBESITY * EPILEPSY	,363	1	,363	5,886	,015	,000	5,886	,679*
OBESITY * YEAR	,405	4	,101	1,642	,161	,000	6,567	,510
OBESITY * UNITSBI	,373	1	,373	6,059	,014	,000	6,059	,692*
EPILEPSY * YEAR	,592	4	,148	2,404	,047	,000	9,615	,696*
EPILEPSY * UNITSBI	,331	1	,331	5,374	,020	,000	5,374	,640*
YEAR * UNITSBI	,440	4	,110	1,784	,129	,000	7,134	,549
Age_r * GENDER * OBES	,095	6	,016	,258	,956	,000	1,549	,121
Age_r * GENDER * EPILEP	,068	6	,011	,185	,981	,000	1,107	,099
Age_r * GENDER * YEAR	,986	24	,041	,667	,888	,000	16,005	,605
Age_r * GENDER * UNITS	,489	6	,082	1,323	,243	,000	7,940	,527
Age_r * OBESITY * EPILEP	,733	6	,122	1,982	,064	,000	11,892	,733
Age_r * OBESITY * YEAR	1,446	24	,060	,978	,492	,000	23,470	,822
Age_r * OBESITY * UNITSBI	,543	6	,090	1,468	,185	,000	8,805	,578
Age_r * EPILEPSY * YEAR	2,599	24	,108	1,757	,012	,000	42,177	,989*
Age_r * EPILEPSY * UNITI	1,003	6	,167	2,713	,012	,000	16,278	,876*
Age_r * YEAR * UNITSBI	1,252	24	,052	,846	,679	,000	20,311	,744
GENDER * OBESITY * EPILEPS	,191	1	,191	3,092	,079	,000	3,092	,420
GENDER * OBESITY * YEAR	,147	4	,037	,595	,666	,000	2,381	,199
GENDER * OBESITY * UNITI	,083	1	,083	1,351	,245	,000	1,351	,213
GENDER * EPILEPSY * YEA	,168	4	,042	,681	,605	,000	2,726	,224
GENDER * EPILEPSY * UNIT	,073	1	,073	1,191	,275	,000	1,191	,194
GENDER * YEAR * UNIT	,277	4	,069	1,124	,343	,000	4,496	,358
OBESITY * EPILEPSY * YEA	,503	4	,126	2,040	,086	,000	8,159	,614
OBESITY * EPILEPSY * UNIT	,365	1	,365	5,923	,015	,000	5,923	,682*
OBESITY * YEAR * UNIT	,165	4	,041	,668	,614	,000	2,673	,220
EPILEPSY * YEAR * UNIT	,436	4	,109	1,770	,132	,000	7,078	,545
Age_r * GENDER * OBESITY								

* EPILEPSY	097	5	,019	,316	,904	,000	1,578	,131
Age_r * GENDER * OBESITY * YEAR	1,326	24	,055	,896	,608	,000	21,515	,776
Age_r * GENDER * OBESITY * UNITSBI	,261	6	,043	,705	,646	,000	4,229	,285
Age_r * GENDER * EPILEPSY * YEAR	1,168	22	,053	,862	,648	,000	18,961	,724
Age_r * GENDER * EPILEPSY * UNITSBI	,250	5	,050	,812	,541	,000	4,059	,296
Age_r * GENDER * YEAR * UNITSBI	1,481	24	,062	1,001	,460	,000	24,035	,834
Age_r * OBESITY * EPILEPSY* * YEAR	1,616	18	,090	1,457	,095	,000	26,221	,915
Age_r * OBESITY * EPILEPSY* UNITSBI	,492	5	,098	1,598	,157	,000	7,989	,564
Age_r * OBESITY * YEAR * UNITSBI	,975	22	,044	,719	,824	,000	15,827	,619
Age_r * EPILEPSY * YEAR * UNITSBI	1,355	20	,068	1,099	,341	,000	21,982	,824
GENDER * OBESITY * EPILEPSY * YEAR	,096	4	,024	,388	,818	,000	1,551	,141
GENDER * OBESITY * EPILEPSY * UNITSBI	,114	1	,114	1,857	,173	,000	1,857	,276
GENDER * OBESITY * YEAR * UNITSBI	,491	4	,123	1,992	,093	,000	7,967	,603
GENDER * EPILEPSY * YEAR * UNITSBI	,241	4	,060	,979	,417	,000	3,917	,314
OBESITY * EPILEPSY * YEAR * UNITSBI	,170	4	,042	,690	,599	,000	2,759	,226
Age_r * GENDER * OBESITY * EPILEPSY * YEAR	,487	10	,049	,790	,638	,000	7,904	,430
Age_r * GENDER * OBESITY * EPILEPSY * UNITSBI	,000	1	,000	,005	,945	,000	,005	,051
Age_r * GENDER * OBESITY * YEAR * UNITSBI	1,992	18	,111	1,796	,020	,000	32,329	,968*
Age_r * GENDER * EPILEPSY * YEAR * UNITSBI	,930	15	,062	1,007	,444	,000	15,099	,680
Age_r * OBESITY * EPILEPSY * YEAR * UNITSBI	,002	1	,002	,025	,875	,000	,025	,053
GENDER * OBESITY * EPILEPSY * YEAR * UNITSBI	,129	1	,129	2,090	,148	,000	2,090	,304

Note: * Observed power of effect (only statistically significant)

Table 5.

Effects of the individual process variables (ARRHYTHMIAS, DYSLIPIDEMIA, and HTN) and of context process variables (YEAR, STROKE UNITS) on the outcome variable (EXITUS). Linear General Model. Full Table

Principal factor	Type III	df		F Quadratic (Pillais)	*p*<.	np2	Centered Parameter	Observ. Power
Individual								
ARRHYTHMIAS	66,003	1	66,003	1056,446	,000***	,006	1056,446	1,00*
DYSLIPIDEMIA	,430	1	43,430	695,140	,000***	,004	695,140	1,00*
HTN	1,151	1	1,151	18,429	,000***	,000	18,429	,990*
ARRHYTHMIAS * DYSLIPIDEMIA	,422	1	,422	6,748	,009**	,000	6,748	,738*
ARRHYTHMIAS * HTN	,437	1	,437	6,987	,008**	,000	6,987	,753*
DYSLIPIDEMIA * HTN	2,656	1	2,656	42,506	,000***	,000	42,506	1,00*
ARRHYTHMIAS * DYSLIPIDEMIA * HTN	,211	1	,211	3,384	,066	,000	3,384	,452
Contextual								
STAY	2,523	3	,841	13,534	,000***	,000	40,603	1,00*
DIAGNOSES	,760	3	,253	4,075	,007**	,000	12,225	,848*
PROCEDURES	6,055	4	1,514	24,366	,000***	,001	97,466	1,00*
READMISSION	,771	1	,771	12,411	,000***	,000	12,411	,941*
STAY * PROCEDURES	1,433	9	,159	2,562	,006**	,000	23,057	,945*
STAY * PROCEDURES	2,752	12	,229	3,691	,000***	,000	44,289	,999*
STAY * READMISSION	1,254	3	,418	6,730	,000***	,000	20,190	,976*
DIAGNOS * PROCEDURES	3,364	12	,280	4,511	,000***	,000	54,137	1,00*
DIAGNOS * READMISSION	,886	3	,295	4,752	,003**	,000	14,257	,902*
PROCEDURES * READMISSION	,767	4	,192	3,088	,015**	,000	12,351	,815*
STAY * DIAGNOS * PROCEDURES	4,469	33	,135	2,180	,000***	,000	71,933	1,00*
STAY * DIAGNOS * READMISSION	1,568	9	,174	2,804	,003**	,000	25,239	,964*
STAY * PROCEDURES * READMISSION	1,345	12	,112	1,804	,042*	,000	21,651	,897*
DIAGNOS * PROCEDUR * READMISSION	2,375	9	,264	4,248	,000***	,000	38,230	,998*
STAY * DIAGNOS * PROCED * READMISSION	2,568	23	,112	1,797	,011**	,000	41,339	,989*

Note: * *p*<.05 *; ** *p*<.01 *** *p*<.001; * Observed power (significant)
